# Refined premature chromosome condensation (G_0_-PCC) with cryo-preserved mitotic cells for rapid radiation biodosimetry

**DOI:** 10.1038/s41598-021-92886-6

**Published:** 2021-06-29

**Authors:** Usha Yadav, Nagesh N. Bhat, Kapil B. Shirsaath, Utkarsha S. Mungse, Balvinder K. Sapra

**Affiliations:** 1 Radiological Physics and Advisory Division, Bhabha Atomic Research Centre, Mumbai, 400085 India; 2 Homi Bhabha National Institute, Anushaktinagar, Mumbai, 400094 India

**Keywords:** Biological techniques, Biotechnology, Cell biology

## Abstract

Mitotic cell fusion induced Premature Chromosome Condensation (G_0_-PCC) assay in human lymphocytes allows rapid detection of cytogenetic damage in interphase stage, within few hours after blood collection. Hence, it is the most suitable method for rapid and high dose biodosimetry. Mitotic cells, used for G_0_-PCC could be either freshly isolated or previously cryo-preserved. However, under emergency scenarios, only cryo-preserved cells can be relied upon, fresh isolation will only delay the process by 18–24 h. Impact of cryopreservation on mitotic cells and their efficacy to induce PCC are not reported. In the present study, we investigated effect of cryopreservation on mitotic cells and refined the parameters for G_0_-PCC. More than 95% of the cells were recoverable after 4 months of cryopreservation, within 20 min recovery at 37 °C, without significant change in the mitotic index or viability. Recovered mitotic cells have shown mitotic index of 89 ± 4% and viability of 90 ± 4%, similar to that of freshly isolated cells. Decrease in metaphases was observed within 40 min after recovery as the mitotic cells progressed through cell cycle and reduced to 21% at 1 h. Nevertheless, in presence of Colcemid, the cells progressed slowly and considerably high metaphase index (60%) persisted up to ~ 2 h. The recovered cells efficiently fused with lymphocytes and induced PCC. Average PCC index varied from 10 to 20%, which did not change with cryopreservation duration. Post fusion incubation duration of 2 h was found to be optimum for proper chromosome condensation. In conclusion, use of cryo-preserved mitotic cells is the most practical approach for rapid biodosimetry. The cells can be recovered quickly and efficiently without alteration in viability or mitotic index. Recovered cells are fully competent to induce G_0_-PCC.

## Introduction

**Figure 1:**
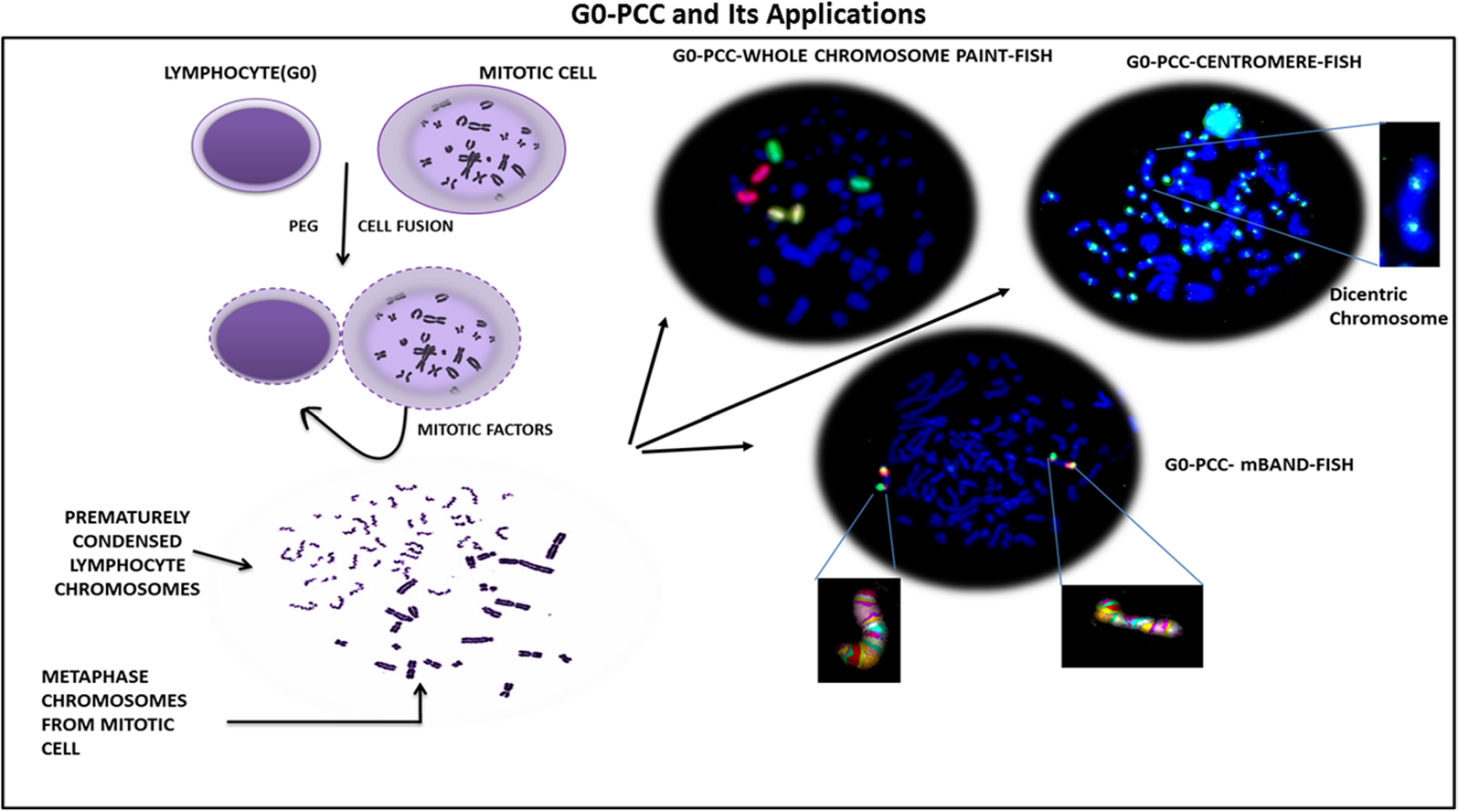
G_0_-PCC and its scopes for chromosomal aberration analysis with applications in FISH.

Chromosomal condensation is a natural process in cell division during the mitotic phase. Condensation of chromosomes is also essential for aberration analysis for various clinical investigations, radiation biodosimetry and other research *purposes*^1–4^. Mitotic cell fusion induced Premature chromosome condensation (G_0_-PCC) assay in human peripheral blood lymphocytes allows rapid detection of cytogenetic damage in interphase stage within few hours after blood collection^5^. Hence, the G_0_-PCC method is most suitable for rapid biodosimetry applications in case of small to large scale radiological *accidents*^6–13^. In addition to biodosimetry, G_0_-PCC has been used to study DNA repair mechanism, radio sensitivity, genotoxicity and cell cycle stages etc.^6,14,15^*.* The method overcomes the shortcomings of conventional standard biodosimetry assays which are based on metaphase chromosomes, needing 48 h of cell culture. Further, conventional methods have limited application in high dose radiation exposures and non-uniform exposures due to radiation induced cell cycle arrest and reduced mitotic index. In combination with Fluorescent in Situ Hybridization (FISH), almost all types of aberrations commonly used in cytogenetic biodosimetry are reported to be quantifiable with G_0_-PCC. For example, excess fragment and ring chromosome with simple uniform Giemsa staining, chromosome specific breaks, inter-chromosomal exchange analysis with whole chromosome painting/multicolour FISH and multicolour banding for intra-chromosomal rearrangement analysis all are feasible with G_0_-PCC (Fig. 1)^12,13^*.*

G_0_-PCC method, however, is tricky and requires a number of optimizations for sufficient fusion yield resulting into scorable PCC spreads. Further, role of PCC is most important during accidental high dose exposures where quick best biological estimate is required for planning the course and timely medical intervention of Acute radiation syndromes or localized tissue injuries in case of non-uniform exposure. It takes around 20 h to collect fresh mitotic cells, which may even extend for several days to a week if large number of mitotic cells are required or if cell culture is not in running condition. Hence, only frozen mitotic cells can be relied upon, as fresh isolation of the mitotic cells will defeat the purpose. However, it is desired that cryopreservation must not affect mitotic cells adversely leading to substantial loss to its efficacy for PCC induction.

Sustained viability of mammalian cells, even decades after cryopreservation is reported. However, loss of viability may occur during preservation/recovery, if optimum conditions are not provided^16–18^. The effect of mitotic cell cryopreservation on its viability, mitotic index, and ability to induce PCC is not well reported. Recovery of mitotic cells from cryopreservation is a critical step, which is also not discussed in literature. Mitotic cells are desired to be available instantly after recollection from cryopreservation, however, necessary recovery time needs to be given for recovering membrane integrity. Further while recovery, mitotic cells may enter into anaphase or cross mitotic phase altogether. Anaphase cells will still induce PCC but the anaphase chromosomes may cause inconvenience while analysing excess fragments of lymphocyte chromosomes. Recoverability in terms of cell number is an important parameter that should remain unaltered but more importantly, the process must not allow the cells to cross through mitotic stage as the metaphase has 10–20 min window in a ~ 24 h cell cycle. In most of the cell line cultures, while reviving the cells, loss of cells up to some extent may not matter much as healthy, viable cells repopulate quickly and dead cells are removed over passage. In contrast, cryo-preserved mitotic cells are to be used immediately, hence their viability and mitotic characteristic to induce PCC are highly important. Further, duration of fusion for PCC should be optimum when chromosomes are condensed enough to score aberrations accurately.

Most of the reports of fusion induced PCC use Chinese hamster ovary cells (CHO) as mitotic cell or sometimes other cell types e.g., HeLa cells. These are very easy to handle and their fast growth allows bulk mitotic cell collection in small culture setup, more importantly, mitotic cells can be isolated with high purity. Additionally, in case of CHO cells, interference of mitotic cell chromosomes in data collection is minimized as they have small number of chromosomes. Further FISH applications using human specific chromosomal probes/paints, mitotic cell chromosomes are excluded altogether.

## Reagents

Dulbecco's Modified Eagle Medium (DMEM), Fetal Bovine Serum (FBS) & Roswell Park Memorial Institute Medium (RPMI1640) were purchased from Gibco, Trypsin, Colcemid, Hanks Buffered Salt Solution, DMSO, Poly Ethylene Glycol w/v 50%, Potassium Chloride, Methanol, Acetic Acid, Calcein AM, Propidium Iodoide (PI) and Giemsa stain were purchased from Sigma. Wheat Germ Agglutinin (WGA) and Hoechst 33,342 dyes were purchased from Thermo-fisher (I34406). DNA probes for human chromosomes for whole chromosome painting, centromere staining, and multicolour banding were purchased from Metasystems.

## Protocol

### Mitotic cell collection

Chinese Hamster Ovary (CHO) cells were procured from National Centre for Cell Science, India. Cells were cultured in DMEM supplemented with 10% FBS. For mitotic cell collection, cells were harvested from a nearly confluent culture, seeded in optimum density of ~ 5 million/175 cm^2^ flask. The cells were allowed to attach and grow for ~ 16 h and then treated with Colcemid (final concentration of 0.2 µg/ml) for ~ 6 h at 37 °C to induce metaphase arrest. The flasks were then kept at − 20 °C for 2–3 min and then mechanically shaken on a hard surface. Detachment of round, loosely attached cells was monitored under inverted microscope. The medium with the cells was collected and centrifuged at 200 g for 8 min.

### Cryopreservation

Mitotic Cell pellet was re-suspended in cryopreservation medium (40% FBS, 10% DMSO in DMEM), counted and transferred to cryo-vials at concentration of ~ 10^6^ cells per ml. Cryovials were then frozen gradually at − 1 °C/min at − 86 °C for 24 h followed by long term storage in vapour phase of LiqN2 at − 196 °C.

### Recovery of cells

The cells which are at mitotic phase tend to move forward with cell cycle, thereby escaping the metaphase, which defeats the purpose of using them for G_0_-PCC. Two methods were attempted in order to revive the cells escaping mitosis. Cryovials with known number of preserved cells were recovered and efficiencies of the two methods were compared. Proportion of dead cells/membrane damaged cells were also compared in the two groups. For this, cell impermeant nuclear dye PI was used, along with live cell permeant nuclear dye Hoechst 33342 to counterstain all the cells. WGA stain was used to mark the plasma membrane of the cells. WGA, Hoechst and PI stocks were prepared in water or PBS and final staining concentration of the dyes was 5 µg/ml, 2 µM and 3 µg/ml respectively. Cells were stained for 10 min at 37 °C.

1. Low temperature (~ 4 °C) recovery: Cryovials were recovered from liq N2 and quickly thawed by adding 4 °C media to the vials. They were then diluted 10 × with cold DMEM medium incubated for 20 min at 4 °C and centrifuged at 200 g for 8 min. The pellets were re-suspended in 4 °C medium and observed for cell recovery.
2. Quick thawing at 37 °C method: Cryovials were recovered from liq N2 and quickly transferred to 37 °C water bath. As soon as the frozen cell suspensions were thawed, they were diluted to 10 × with pre-warmed media containing 0.2 µg/ml Colcemid. An incubation of 20 min was provided at 37 °C for membrane damage recovery. The suspension was then centrifuged at 200 g for 8 min and the cell pellet was re-suspended into fresh medium containing Colcemid. Escape of mitotic cells from metaphase with time of recovery was observed by chromosomal spread preparation of cells recovered in presence and absence of Colcemid.

### Viability test

Recovered cryo-preserved or freshly isolated mitotic cells were re-suspended in PBS and stained with Calcein-AM for 15 min in PBS followed by 10 min PI staining. After staining, cells were transferred onto clean glass slides and observed under the microscope. Cells with green fluorescence were considered as live while ones with red were dead cells. For each set, total 400–600 cells were counted. Proportion of live cells among the total was plotted against duration of cryopreservation.

### Mitotic index assessment

After recovery from cryopreservation, cells were counted. 0.5 M cells were centrifuged at 200 g for 8 min and re-suspended in 0.45% KCl hypotonic solution for 6 min followed by smooth addition of 1 ml fixative (3:1 methanol: Glacial acetic acid) before centrifugation. Cells were then washed three times with fixative before slide preparation. Finally, cell pellets were suspended in ~ 50 µl of fixative and dropped from 2 cm height onto a clean wet glass slide which was just taken out from chilled distilled water. The spreads were heat fixed at 37 °C on a hot plate. Slides were stained with 4% Giemsa stain for 10 min and scored manually under a microscope. Each chromosomal spread was scored as mitotic cell and un-burst nuclei as non-mitotic cell. On an average, more than 500 cells were counted for each data point to calculate % mitotic index (proportion of mitotic cells among total cells).

### Blood collection

Fresh Blood samples were collected from healthy consented volunteers in lithium heparin coated vials.

### Lymphocyte isolation

Blood samples were diluted 1:1 in RPMI1640 medium. Diluted blood was layered over the top of Ficoll reagent in equal volumes in 15 ml centrifuge tubes, followed by centrifugation at 400 g, 30 min, 25 °C. Buffy coat was isolated and washed with RPMI1640 medium twice.

### Cell fusion

Mitotic cells were mixed with isolated lymphocytes in a ratio of 1:5 (0.4 M: 2 M), the pellet was re-suspended in 80 µl of PEG for 2 min and mixed manually. The pellet was then diluted with 4 ml RPMI and centrifuged. The cells were then incubated for PCC at 37 °C followed by fixation and slide preparation as described for CHO mitotic cells.

### Fusion/PCC index assessment

Slides were stained with Giemsa stain and scored manually under microscope. Chromosomal spreads with both metaphase chromosomes of mitotic cells and prematurely condensed G_0_-Phase chromosomes from lymphocyte were counted as PCC spread. Frequency of PCC out of total chromosomal spreads was calculated as PCC index. Three independent fusion experiments with scoring of 500–1000 spreads in each experiment were carried out for calculation of average PCC index.

### Fusion quality assessment

Chromosomal spreads from varied post-fusion incubation periods (0, 30 min, 1 h, 1.5 h and 2 h) were analysed. 200 PCC spreads for each sample were scored and categorized into three classes viz., poor, moderate and well based on visual PCC levels.

### FISH

Protocols from the Metasystem Probes, the manufacturing company, were followed.

### Image capturing and data analysis

Images were captured using Axioimager-Z2 microscope with auto-capture modules from Metasystems, with the help of Metafer-5, IKAROS and ISIS software. Scoring of samples was done manually. On an average more than 500 cells per sample were counted in each experiment for testing viability, mitotic index and PCC index from cryo-preserved mitotic cells. Mean values were plotted with Poisson error at 95% confidence interval. Data analysis was done with Excel or Origin 8.0.

## Results and discussion

### Effect of cryopreservation on recovery of mitotic cells

**Figure 2:**
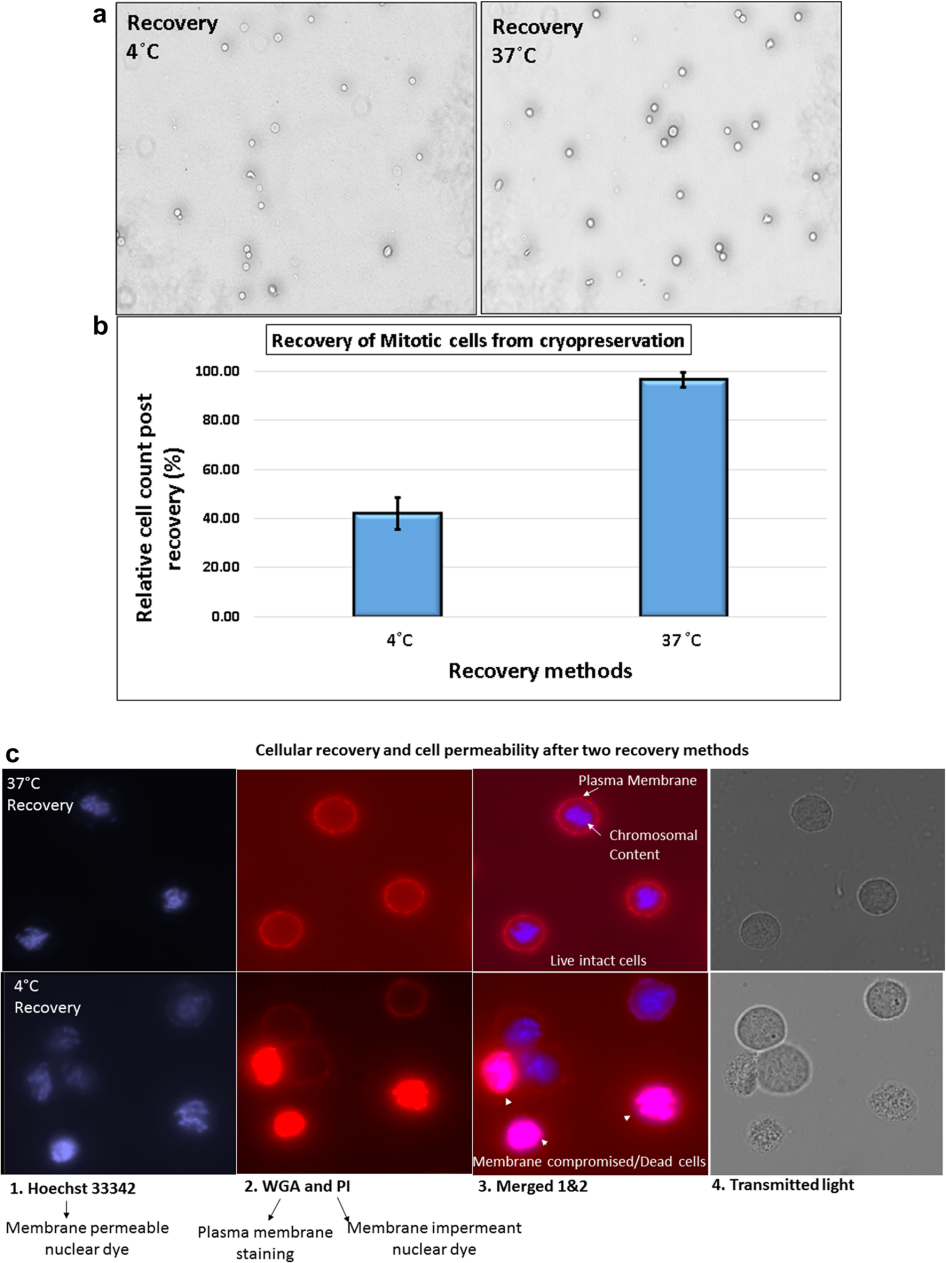

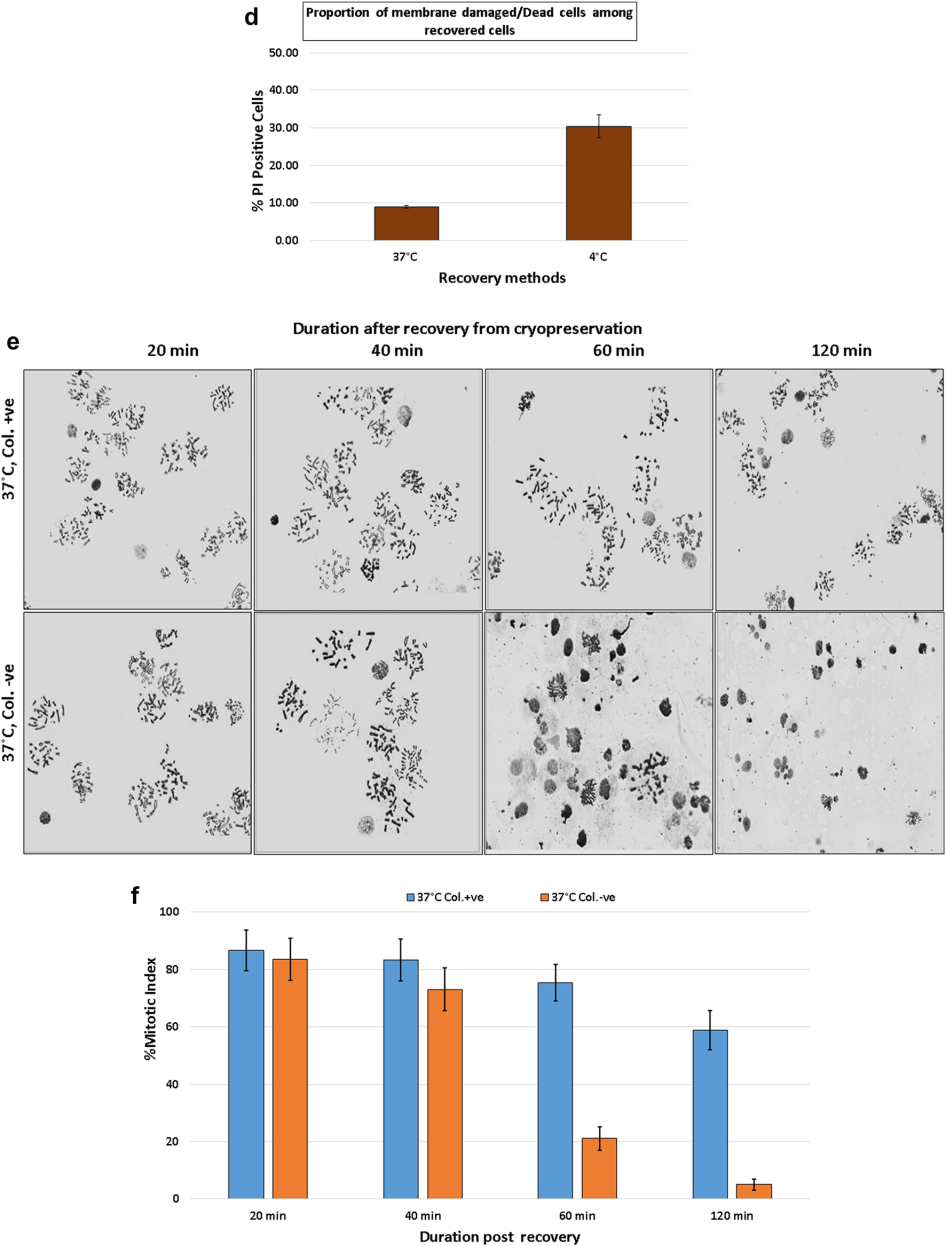
(**a**) Microscopic view of the cells from twin vials which were recovered from cryopreservation using two different methods: at 4 °C (top left) and at 37 °C in presence of Colcemid (top right), (**b**) quantitaive data of cell recovery with the two methods. (Error bar represents Mean ± SD from three independent experiments), (**c**) Damaged and live cell nucleus staining using Hoechst 33342 and PI. PI positive red/pink nuclear content represent dead cells or cell with compromised membrane integrity and blue nuclear content suggest live intact cell. Plasma membrane was marked using WGA staining visible in live intact cells. (**d**) Quantitative data of PI positive cells among the recovered cryopreserved cells using the two recovery methods (Error bar represents Mean ± SD from three independent experiments). (**e**) Progression of recovered mitotic cells from metaphase in presence and absence of Colcemid. (**f**) Quantitative data of kinetics of mitotic index in absence and presence of Colcemid after recovery from cryopreservation (Error bar represents Mean ± 1.96SE).

Post-cryopreservation, recovery methods were aimed to recover maximum proportion of the preserved cells without allowing cell cycle progression beyond mitosis, either by using Colcemid or with application of low temperature. Vials with known numbers of mitotic cells were cryo-preserved for 4 months before the experiment and recovered by two approaches as described in the methods. Out of the two recovery methods, quick thawing at 37 °C in presence of Colcemid resulted in more than 90% of cells recovery among three tested vials whereas, in the 4 °C thawing method, ~ 50% cells were lost. Morphology of the cells also appeared to be better in case of quick thawing at 37 °C (Fig. 2a–c). Morphological features of dead cells, like, irregular shape, faint appearance was observed in case of 4 °C recovered cells, whereas cells recovered at 37 °C were bright and round similar to that of freshly isolated mitotic cells. Further, live cell impermeant nuclear dye PI was used among the recovered cells to stain dead cells and damaged cells (cells with compromised membrane integrity). Along with PI, counterstaining of all the cells was performed using live cell permeable nuclear dye Hoechst 33,342. PI positive cells were counted as damaged/ dead cells among all counterstained cells (Fig. 2c). Proportion of these cells was found to be considerably high, ~ 30% among 4 °C compared to ~ 9% among 37 °C recovered cells (Fig. 2d). It may be noted that overall live cell recovery was < 35% at 4 °C compared to ~ 90% at 37 °C. Possible reasons for loss of cells at low temperature thawing could be ice crystal formation leading to rupture of cells. Further, membrane recovery at cold temperature may be impaired. These factors may cause lysis of cells prior or during centrifugation process. All subsequent experiments were performed only for the recovery method of 37 °C.

Further, mitotic cells recovered in absence of Colcemid at 37 °C tend to move through cell cycle quickly. It is known fact that total duration of mitotic phase is typically only about 1 h. Considerable number of cells enter into late mitotic phases and cross cell cycle altogether or undergo mitotic catastrophe within 1 h (Fig. 2e). Kinetics of mitotic cells in absence and presence of Colcemid was assessed with increasing recovery time (20 min, 40 min, 60 min and 120 min). Mitotic index in presence of Colcemid was found to be ~ 87% after 20 min which slowly decreased to 83%, 75% and ~ 60% at 40 min, 60 min and 120 min respectively. Among the mitotic cells, proportion of late anaphases was less than 5% up to 60 min and ~ 7% at 120 min. Mitotic index in absence of Colcemid was about 83% at 20 min and decreased drastically to 75%, 21% and 5% at 40 min, 60 min and 120 min respectively (Fig. 2f). Further, at 40 min where mitotic index was 75%, about 23% of the mitotic cells were late metaphases. Hence, it is advisable that mitotic cells should be used as soon as possible after membrane recovery and cells should be recovered in presence of Colcemid.

Further viability and mitotic index were assessed with cells recovered after variable duration of cryopreservation by quick thawing at 37 °C for 20 min in presence of Colcemid.

## Effect of cryopreservation on viability, mitotic index and fusion ability

### Viability

**Figure 3:**
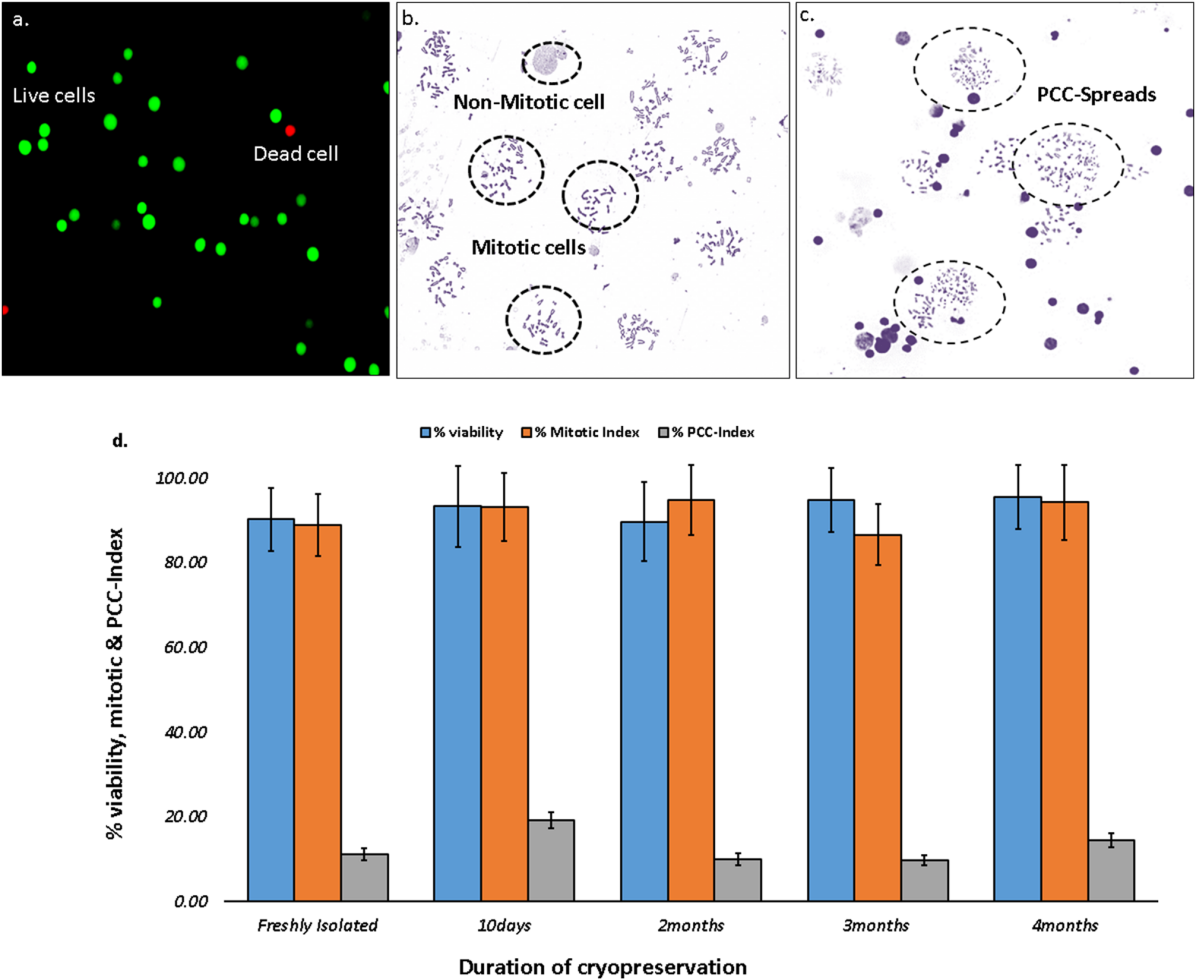
Evaluation checkpoints of cryopreservation mitotic cells for PCC (**a**) Viability; Mitotic cells after differential staining of live dead cells (**b**) Mitotic index; Chromosomal spreads from mitotic cells in a microscopic view. Each chromosome spread represents one mitotic cell (encircled) and nuclei which did not form spread counted as non-mitotic cells. (**c**) Fusion/PCC index Chromosomal spread preparation on slide after cell fusion induced PCC. PCC spreads can be identified by presence of both metaphase chromosomes from mitotic cell and prematurely condensed chromosomes from lymphocytes (encircled). (**d**) Viability, mitotic index and PCC index of mitotic cells after variable duration of cryopreservation. (Error bar represents 1.96xSE of Mean).

Live and dead cells were counted microscopically after differential live-dead staining with vital dye Calcein AM and exclusion dye PI. Live cells showed green fluorescence and dead cells showed red fluorescence. Average viability of freshly collected mitotic cells was 90.2 ± 3.8%. Cells with variable duration of cryopreservation (10 days, 2, 3 and 4 month) showed no significant difference from that of freshly collected unpreserved cells (Fig. 3a, d).

As mentioned earlier, it is preferred to cryopreserve these mitotic cells for instant availability at the time of requirement. In this study, we have demonstrated that cryopreservation and proper recovery method does not affect viability of the preserved mitotic cells for at least up to 4 months. This duration may be considered as most useful and practical duration of storage for the described purpose by any lab, aiming such applications. Sustained viability of mammalian cells, years to decades after cryopreservation, is also reported^17^. Hence, along with evidence of our results, we believe that, even duration longer than four months may not affect the viability for mitotic cells as well, if proper gradual freezing and quick recovery methods are adapted.

### Mitotic index

Mitotic index among mitotic cells was also found to be in the same range as viability. Freshly isolated cells without any preservation have shown mitotic index of 90 ± 3.7%. Mitotic index in cryo-preserved cells did not vary considerably from that of freshly isolated cells. The purity of mitotic cells remained unaltered during the 4 months course of cryopreservation. Mitotic index and viability in all the sets ranged from 87 to 95% and 90–95% respectively (Fig. 3b, d). For successful PCC, it is important that cells are viable and mitotically active. If CHO cells are not in mitosis, fusion will not yield PCC. Hence, it is desirable to have maximum possible metaphase index. Corroborating our results^19^, also reported mitotic index of 95% for freshly isolated mitotic CHO cells. However, we could not find reference to compare results of cryo-preserved mitotic cells. The mitotic index discussed here was scored in the cells fixed 20 min after recovery from cryopreservation. Observations on effect of recovery duration and condition on the metaphase index has been discussed in the previous section on cellular recovery.

### PCC index

With establishment of sustained viability and mitotic index of cryo-preserved mitotic cells, our next objective was to ensure if they were capable of fusion with lymphocytes and inducing PCC in the same way as freshly isolated cells. Mitotic cells were mixed with lymphocytes in a ratio of 1:5 and fused with the help of 50% PEG treated for 2 min, as per the described procedure. In the mixed cell suspension, there are possibilities of all combinations of cell fusions; mitotic cells with mitotic cells, mitotic cells with lymphocytes, and lymphocytes with lymphocytes, however, our particular interest is in the proportion of mitotic cells fused to lymphocytes to induce PCC. Chromosomal spreads with both mitotic CHO and G_0_-phase lymphocyte chromosomes were identified as PCC spreads. Frequency of PCC spreads per 100 metaphase spreads was defined as PCC index. In our experiments PCC index ranged from 6 to 32%. With multiple experiments, average frequency was found to be 10–20%. We could not find any pattern associated with duration of cryopreservation (Fig. 3c, d). Inter-experimental variation is reported in cell fusion processes and the cause is not well understood. Some known reasons for variation of PCC index of fusion include, variation in the cell ratios, PEG concentration, duration of PEG treatment etc. Despite fixed parameters, variability could be possible due to variation in microenvironment in the suspension during fusion such as variation in manual mixing of PEG with cells, slight variation in treatment time, temperature, volume of suspension etc. Nonetheless, PCC Index of 5% or more can be considered efficient for biodosimetry applications as it yields sufficient number of PCC spreads for microscopy on a single slide.

There are a few reports on PCC index as provided further, however, use of cryo-preserved mitotic cells is not reported. Pantelias and Maillie^19^, in a detailed study of cell fusion indices in G_0_-PCC, reported 8.7 PCCs per 100 metaphases with 50% PEG concentration. However, we slightly tweaked the protocol by using 1400 MW hybridoma tested PEG and lymphocyte to mitotic cell ratio of 5:1 compare to 1000 MW PEG and ratio of 6.5:1 respectively used by Pantelias and Maillie^19^.

Neronova^20^ also reported a PCC index of 3.6 per 100 lymphocytes, which would correspond to ~ 18% PCC index per 100 mitotic cells by considering their 1:5 cell ratio of mitotic cell to lymphocytes. The index is not unusual and lies within our experimental results. However, it could be an overestimate as index is based on counting PCCs per lymphocyte. Counting of mitotic cells and PCC spreads is simple with distinct appearance of chromosomal spread. However, as explained by^19^*,* counting of interphase lymphocyte cells could be erroneous and underestimated at high concentrations of PEG (50–55%) due to clumping of cells, finally leading to overestimation of PCC index. Moreover, both the reports support our results.

## Whole chromosome painting: demonstration of multi-cell fusion

**Figure 4:**
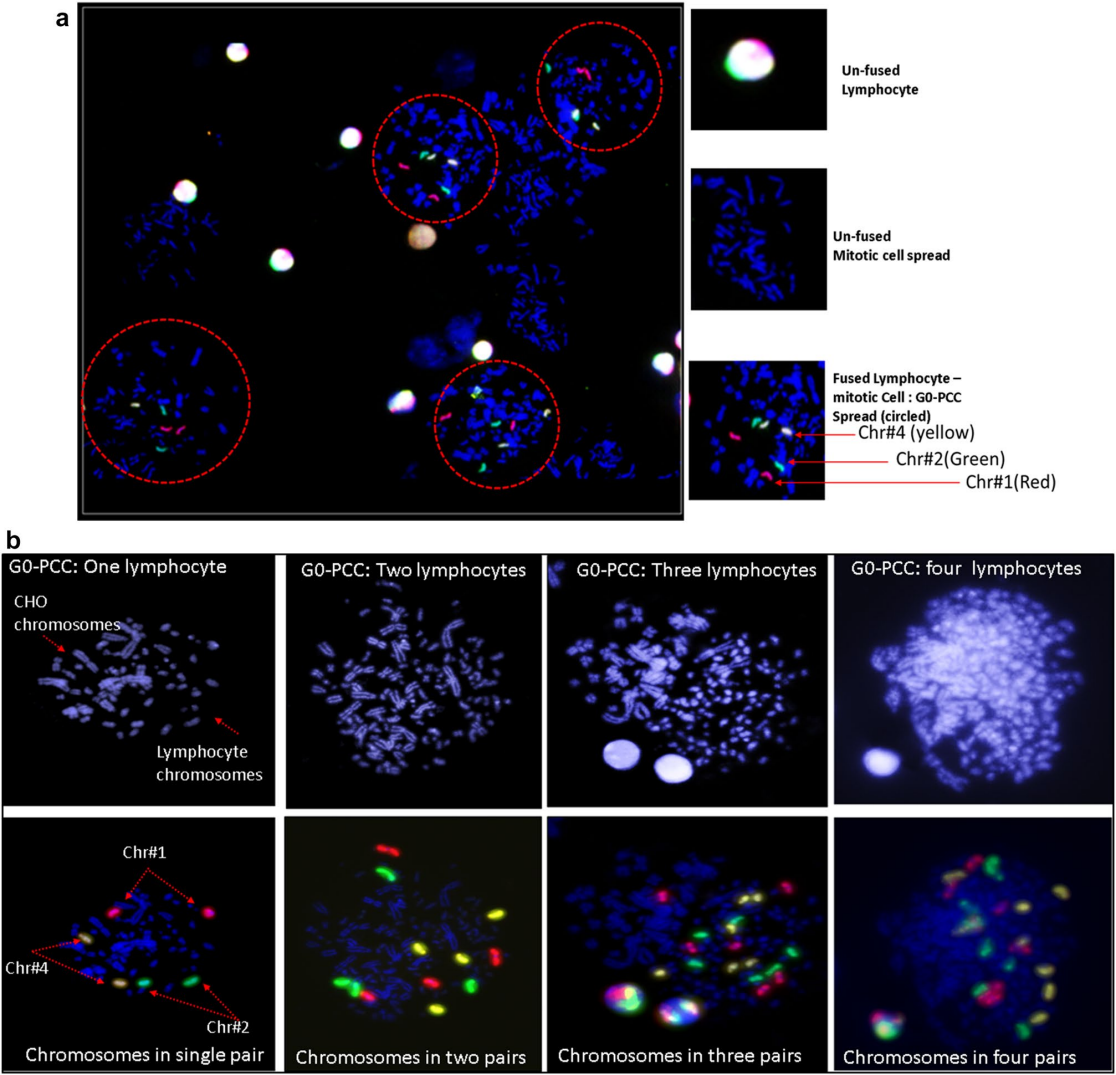
(**a**) A typical field of chromosome spread preparation G_0_-PCC FISH slide showing efficient fusion yield. PCC spread can be identified by the presence of painted human chromosome pairs 1, 2 and 4 in red green and yellow respectively (encircled), (**b**) G_0_-PCC spreads from one, two, three or four lymphocyte fusion to mitotic cell/s. Increase in number of lymphocyte chromosomes and size of the spread can be observed (top row, left to right). Multichannel view of spread shows red green and yellow fluorescent signals from painted chromosomes 1, 2 and 4, respectively. Since one lymphocyte caries one pair of each chromosome, presence of one, two, three and four pairs of each of the painted chromosomes confirm fusion of one two three and four lymphocyte respectively (left to right bottom row).

For visual demonstration of efficient PCC induction, we have performed whole chromosome painting of human chromosomes 1, 2 and 4 in red, green and yellow respectively with single cocktail of FISH probes. Chromosomal spreads with three colours of painted chromosomes represent G_0_-PCC spread and spreads without painting represent mitotic cell alone (Fig. 4a). Each lymphocyte has one pair of each chromosomes hence, two fluorescent signals for each painted chromosome can be seen in a PCC spread. In an efficient fusion process, occasionally multi-cell fusions also occur where more than one lymphocytes are fused with one or more mitotic cells. With whole chromosome painting, we have demonstrated the PCC spreads with one, two, three or four lymphocytes fused with mitotic cell/s. Since one lymphocyte has two signals of each chromosome, number of 4, 6 or 8 signals for any painted chromosome was identified as two, three and four lymphocyte fusion (Fig. 4b). The multi cell fusions are not desirable as spreads have more chances of chromosomal overlaps and loss of chromosomes during multiple fusion. However, they occur quite rarely, hence, can be ignored in case aberrations are not scorable. After scoring more than 600 PCCs, frequency of two lymphocyte fusions was found to be around 5% whereas frequency for three lymphocyte fusions was 0.6%.

## Gradual premature condensation of chromosomes with increasing post fusion incubation duration

**Figure 5:**
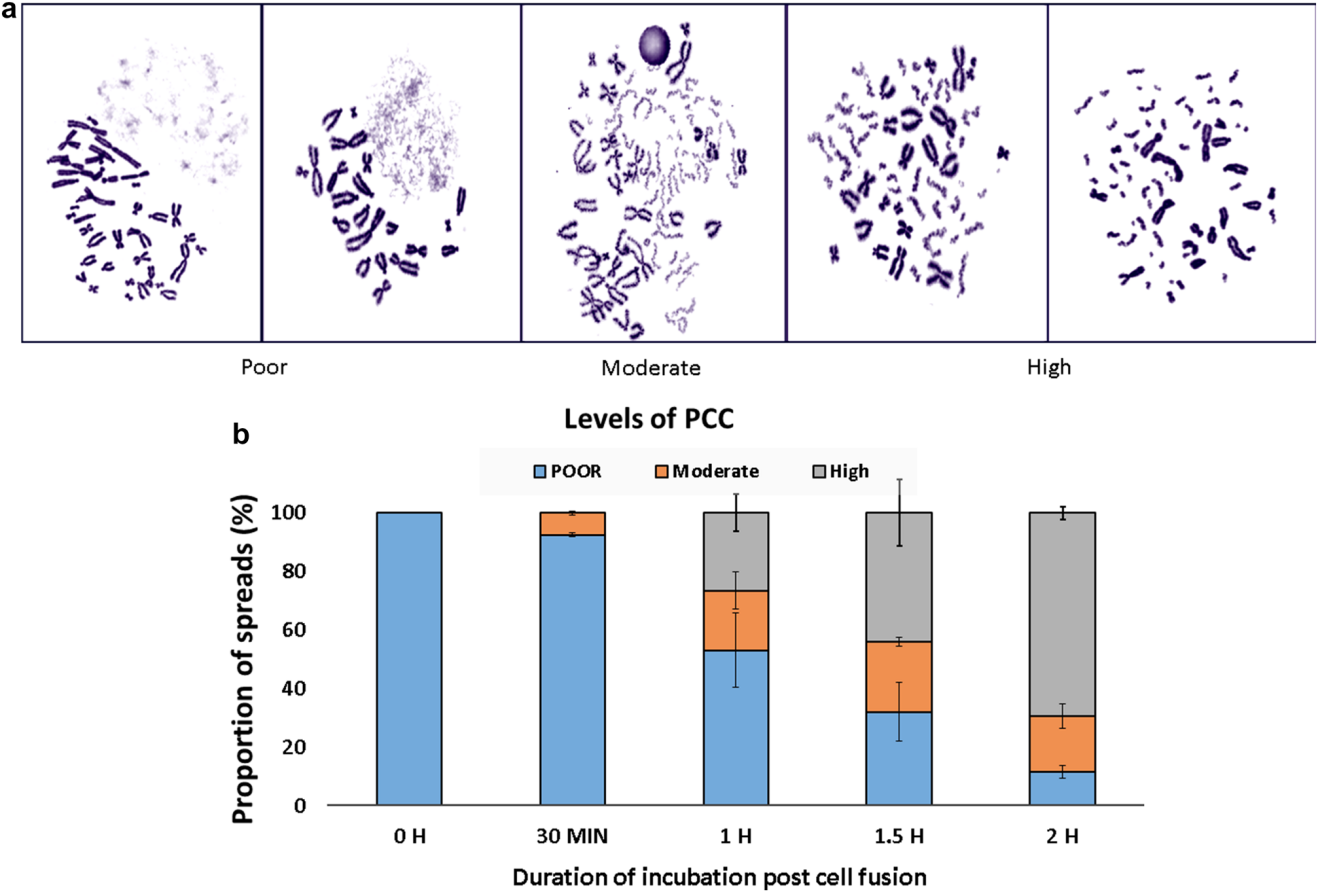
(**a**) Levels of chromosome condensation in PCC from 0 to 2 h after cell fusion. (**b**) Quantification of proportions of poor, moderate and well condensed spreads.

Condensation level of chromosomes decides the quality of PCC-spreads, which in turn, can influence chromosomal aberration quantification. The condensation occurs during post fusion incubation and hence, the duration of this incubation can influence the level of condensation. We studied impact of post fusion incubation time on spread quality. After the cell fusion, incubation duration of 0 h, 0.5 h, 1 h, 1.5 h and 2 h was allowed for chromosomes condensation. Condensation levels among different spreads were categorized in three broad classes; poor, moderate and high. It was observed that the level of condensation increased with time (Fig. 5a). When spreads were prepared immediately after the fusion, due to poor condensation, lymphocyte chromosomes have shown indistinct morphology. Spreads with different levels of condensation were scored against post fusion incubation time (Fig. 5b). At longest incubation time tested i.e., 2 h, ~ 70% spreads were observed to be well condensed. Moderately condensed chromosomes are longer and show discontinuity in the staining which could be mistaken as a chromosomal break. Inclusion of these spreads could result in errors in counting excess breaks. Higher levels of condensations ease the counting of fragments. Though further increase in the post fusion incubation duration may result in further improvement in frequency of well condensed chromosomes, it will also result in unnecessary delay as well as excess condensation of chromosomes. Hence, we consider incubation duration of 2 h as optimum for excess fragment counting. Pantelias and Maillie^19^ reported gradual condensation of chromosomes from 15 to 90 min qualitatively, however, quantitative data was not provided.

## Conclusions

Cryo-preserved mitotic cells, which are preserved over several months, can be efficiently utilised for induction of PCC in G_0_-phase human lymphocytes. Mitotic cells can be recovered from cryopreservation efficiently within 20 min. It was found that cryopreservation did not alter viability, mitotic index and fusion capability of mitotic cells, at least up to four months of preservation duration. It is also concluded that, to induce PCC in fused cells, post incubation duration is crucial and 2 h of incubation time is optimum for PCC to induce well condensed spreads in high frequency.

### Ethics approval

All the work carried out with approval of Institutional committee, “Medical Ethics Committee, BARC Hospital, Bhabha Atomic Research Centre”. All the methods were performed in accordance with the relevant guidelines from the committee. Informed consents were obtained from adult healthy volunteers before blood sample collection.

### Consent to participate

Informed consents were obtained from individual participants in the study.

## Data Availability

Available from the corresponding author on reasonable request.
